# Metabolic rate, context‐dependent selection, and the competition‐colonization trade‐off

**DOI:** 10.1002/evl3.174

**Published:** 2020-06-12

**Authors:** Amanda K. Pettersen, Matthew D. Hall, Craig R. White, Dustin J. Marshall

**Affiliations:** ^1^ School of Biological Sciences/Centre for Geometric Biology Monash University Melbourne VIC 3800 Australia; ^2^ Department of Biology Lund University Lund 221 00 Sweden

**Keywords:** Fecundity, fertility, fitness, growth, interspecific competition, intraspecific competition, longevity, metabolism, pace‐of‐life, viability, reproduction, larval size

## Abstract

Metabolism is linked with the pace‐of‐life, co‐varying with survival, growth, and reproduction. Metabolic rates should therefore be under strong selection and, if heritable, become less variable over time. Yet intraspecific variation in metabolic rates is ubiquitous, even after accounting for body mass and temperature. Theory predicts variable selection maintains trait variation, but field estimates of how selection on metabolism varies are rare. We use a model marine invertebrate to estimate selection on metabolic rates in the wild under different competitive environments. Fitness landscapes varied among environments separated by a few centimeters: interspecific competition selected for higher metabolism, and a faster pace‐of‐life, relative to competition‐free environments. Populations experience a mosaic of competitive regimes; we find metabolism mediates a competition‐colonization trade‐off across these regimes. Although high metabolic phenotypes possess greater competitive ability, in the absence of competitors, low metabolic phenotypes are better colonizers. Spatial heterogeneity and the variable selection on metabolic rates that it generates is likely to maintain variation in metabolic rate, despite strong selection in any single environment.

**Impact Summary**

The rate at which organisms uptake, transform, and expend energy varies substantially across individuals of the same species, even after accounting for differences in body size and temperature. What drives this variation? Metabolic rates set the pace‐of‐life—higher metabolic rates are linked to faster growth, earlier onset of reproduction, and shorter life span, whereas low metabolic rates are associated with a slow pace‐of‐life (slow growth, late onset of reproduction, and long life span). Hence, variation in metabolic rates are likely to have fitness consequences, and be under strong selection. Evolutionary theory predicts that over time, selection should deplete variation in traits, yet variation in metabolic rates is ubiquitous. Alternatively, variable selection regimes may maintain trait variation, by selecting for different metabolic rates across different environments, where a high or low pace‐of‐life is advantageous. Although this theory is well established, field estimates of selection on metabolism across environments are historically rare. To investigate the role of environmental variation in maintaining trait variation, we measured the metabolic rates of individual marine bryozoans, experimentally manipulated their competitive environment, and monitored their survival, reproduction, and pace‐of‐life in the subtidal. We found that selection on metabolic rate varies among competition environments separated by only a few centimeters—competition selects for a faster pace‐of‐life, relative to competition‐free environments. High‐metabolism individuals are better able to withstand intense competition; however, low‐metabolism individuals live longer and are likely to have higher fitness under competition‐free conditions. Hence, the environment‐dependent nature of selection on metabolism and the pace‐of‐life is likely to maintain variation in metabolic rates.

Metabolic rate reflects the pace at which organisms use, transform, and expend energy to sustain life. Metabolism covaries with components of fitness such as survival, growth, longevity, and reproduction (Glazier 2005; Auer et al. 2018b; Pettersen et al. 2018). Over the last century, metabolic theory has explored the origin and maintenance of metabolic scaling relationships from single‐celled organisms to communities (Rubner 1908; Kleiber 1932; Hemmingsen 1960; Kooijman 2000; Gillooly et al. 2001; Brown et al. 2004; White et al. 2008; Glazier 2010). Historically, studies of metabolism have emphasized mechanistic models of physical constraints to explain variation in metabolic rate, but recent evidence suggests that multivariate selection shapes much of the among‐species variation in metabolic rate across macroevolutionary scales (White et al. 2019). The action of selection across broad, evolutionary timescales may explain macroevolutionary patterns in metabolic scaling, but the nature of selection on metabolism at shorter time scales is much more ambiguous. Within species, considerable variation in metabolism persists—basal and standard metabolic rates vary up to threefold within species, even after holding mass and temperature constant (Burton et al. 2011; Konarzewski and Ksiazek 2013; White and Kearney 2013). Theories focused on mechanism struggle to account for this variation; so we look to evolutionary theory to understand the context‐dependent nature of selection acting on metabolic rates (Pettersen et al. 2018).

A classic tenet of evolutionary theory is that selection depletes genetic and (or) phenotypic variation, such that heritable traits can become less genetically variable over time (Arnold et al. 2001; Merilä et al. 2001; Wilson et al. 2006). Metabolic rates are both heritable and subject to selection (Garland and Carter 1994; Pettersen et al. 2018; White et al. 2019), yet vary within populations and among individuals of the same species. Natural environments can fluctuate across spatial and temporal scales, and so too can selection (Bell 2010; Charmantier et al. 2014; Lange et al. 2016). Fluctuating selection can maintain trait variation (McDonald and Ayala 1974; Calsbeek et al. 2010; Bertram and Masel 2019), and may explain why we observe intraspecific variation in metabolic rates (Sasaki and Ellner 1997). A continually shifting environment where different phenotypes are favored under different conditions should maintain phenotypic variation. Accordingly, studies generally show that selection can vary substantially in space and over time, even over very small scales (meters and days; Grant and Grant 2002; Svensson and Sinervo 2004; Siepielski et al. 2009; Whitman and Agrawal 2009; Bell 2010; Burton et al. 2011). Variation in selection regimes could maintain intraspecific genetic, and therefore phenotypic variation, in metabolic rate, yet formal estimates of selection on metabolism across natural field conditions are rare.

Competition is a ubiquitous and powerful agent of selection in nature. Both intra‐ and interspecific competition are important eco‐evolutionary processes, affecting individual access to resources that can ultimately drive evolutionary change (Fussmann et al. 2007). Variation in the form and intensity of competition can arise due to differences in densities of individuals or the relative abundance of shared resources, creating competition‐dependent selection regimes across time and space. Even within a single population, individuals can experience very different levels of intra‐ and interspecific competition, with profound consequences for fitness (Wissinger 1989; Stratton 1995; Einum et al. 2008; O'Neal and Juliano 2013). Hence, the presence, strength, and form of competition are likely to vary across environments, to produce spatially explicit selection regimes.

Competition is particularly likely to alter selection on metabolic rates. Competition alters the supply of, and access to, resources to influence metabolic rates and the pace of the life history in unexpected ways (Marshall 2005; DeLong et al. 2014; Bassar et al. 2016; Ghedini et al. 2018). High‐population densities generally reduce per capita resources. A fast pace‐of‐life has been associated with dominance over conspecifics for food and territory, greater foraging efficiency, and faster digestion—hence, individuals with higher metabolic rates may be more competitive if they are better able to acquire and assimilate resources so that they may reach a size refuge or outcompete conspecifics (Mueller and Diamond 2001; Burton et al. 2011; Nilsson and Nilsson 2016; Auer et al. 2018b). Alternatively, lower metabolic phenotypes with relatively low resource requirements may be beneficial for preserving energy reserves and resisting starvation (Ghedini et al. 2017). The competition environment should thus interact with metabolic rates, and perhaps alter the costs and benefits of a particular metabolic phenotype (Swanson et al. 2017). It seems plausible, therefore, that variation in competitive environments may mediate selection on metabolic rates and maintain intraspecific variation more generally.

Here, we measure how competition alters selection on metabolic rates in the field in the marine bryozoan *Bugula neritina*. *Bugula neritina* produces free‐swimming offspring that typically disperse centimeters to hundreds of meters, and can experience a range of competition environments, from newly disturbed free space to densely packed communities (Marshall and Keough 2009). We leverage the tractability of this system to experimentally manipulate the competitive environment of individuals of known metabolic phenotypes and monitor their survival, fertility, and reproduction in the field. We then formally estimate a series of parameters related to selection (i) the opportunity for selection (*I*) across competition levels; (ii) linear (*β*) and nonlinear (*γ*) selection gradients; and (iii) the intensity of selection (*V*). Finally, we (iv) measure the covariance between metabolic rates and key life‐history traits—growth rate, longevity, and age at onset of reproduction. Combined, measures of form, opportunity, and intensity of selection allow us to quantify selection across environments that are needed to reveal the complex interplay of phenotype, fitness, and the pace‐of‐life driving natural variation in metabolic rates (Brodie et al. 1995; Hall et al. 2008). We find that metabolic rates mediate a trade‐off between colonization and competition—high‐metabolism individuals were better able to withstand intense competition but low‐metabolism individuals lived for longer and are likely to have higher fitness under competition‐free conditions.

## Materials and Methods

### STUDY SPECIES AND FIELD DEPLOYMENT

*Bugula neritina* (named by genus hereafter) is a filter‐feeding, arborescent bryozoan that inhabits a range of shallow subtidal surfaces, including boat hulls and pier pylons (Chang and Marshall 2016). *Bugula* colonies brood fertilized eggs in visible reproductive structures (ovicells) for approximately one week (Woollacott and Zimmer 1975). Light at dawn induces spawning of free‐swimming, lecithotrophic larvae that spend a short time in the plankton, typically settling within minutes to hours (Marshall and Keough 2003). Settlers then undergo metamorphosis over approximately three days to develop, and form the ancestral zooid (Burgess and Marshall 2011). The ancestral zooid then begins feeding and grows into a colony via asexual budding, and reaches sexual maturity approximately 3–8 weeks postsettlement.

All field collections and monitoring were conducted at Royal Brighton Yacht Club, Victoria, Australia (–37.909, 144.986) during March to September 2015. Ten sexually mature colonies were transported back to the laboratory and kept in a dark controlled temperature room for two days. Colonies were then placed into a single beaker containing filtered seawater, exposed to light and induced to spawn following standard procedures (Pettersen et al. 2016). A single *Bugula* colony releases hundreds to thousands of free‐swimming larvae—we took a random subsample of “focal” individuals for measuring the traits of interest (see below). Each focal individual, attached to a small piece of acetate, was then glued onto a labeled PVC plate (55 × 55 × 3 mm; our unit of replication) and randomly assigned to one of three treatments: no competition (“*nocomp*”), intraspecific competition (“*intra*”), and interspecific competition (“*inter*”; Fig. S1).

Individuals in *nocomp* were glued onto a blank plate, and biofouling kept clear throughout the duration of the study. The *intra* treatment represents an environment of intense intraspecific competition—commonly experienced by *Bugula* in the field (Allen et al. 2008). The focal individual (for which measurements were taken) was glued onto a plate among eight individual *Bugula* settlers of the same age. Throughout monitoring, plates were cleared of any exogenous settlers of any species. The *inter* environment mimicked settlement into a pre‐established, subtidal community. To obtain these communities, we left the plates in the field for 12 weeks prior to starting our experiment to allow natural fouling onto these plates. Upon return to the laboratory, a small area (15 × 15 mm) in one corner of the plate was cleared of any organisms, mimicking a physical disturbance, and the focal individual glued into this section. Once all individuals were introduced into their treatments, plates were maintained overnight in tanks with unfiltered seawater before being deployed the next morning onto PVC backing panels (570 × 570 × 6 mm) in the subtidal as per Pettersen et al. (2016). Overall, our experiment included 360 individuals of known phenotype outplanted on individual plates (120 per competition treatment) that were randomly assigned across 10 backing panels (36 plates per panel, 12 from each treatment). Each backing panel was deployed in the subtidal, suspended at 1.5‐m depth at 5‐m intervals along the length of a single pontoon at Royal Brighton Yacht Club.

### TRAIT AND FITNESS MEASUREMENTS

#### Traits of interest: Larval mass and metabolic rates

We measured selection on three traits: larval mass, and metabolic rate at two stages during early ontogeny: two hours postsettlement and 24 hours postsettlement (hereafter referred to as metabolic rate early (MR_E_) and metabolic rate late (MR_L_)). MR_E_ and MR_L_ occur during crucial stages during the life history and have previously been shown to be under differing selection in this species (Pettersen et al. 2016). Larval mass is a key life history trait and a well‐known predictor of performance; however, the offspring size‐performance relationship is often context dependent (Marshall et al. 2018). We measured the diameter of newly spawned larvae to the nearest μm and calculated larval mass (μg) using previously developed protocols (Pettersen et al. 2015). Metabolic rate was measured for individuals using the common proxy, rate of oxygen consumption or V̇O_2_, as per previous methods (Pettersen et al. 2015). In summary, individuals were settled in a drop of seawater onto small sheets of acetate and placed into glass vials containing pasteurized, 0.2‐μm‐filtered seawater and a nonconsumptive O_2_ sensor spot. We used 24‐channel PreSens sensor dish readers (Sensor Dish Reader SDR2, PreSens, Germany) with 24‐chamber 200 μL glass microplates (Loligo Systems Aps, Tjele, Denmark) to measure V̇O_2_ (rate of change of O_2_ saturation over time; %h^−1^), where readings were taken every 2 min over 3‐h intervals. We then used the package “LoLinR” to objectively and reproducibly estimate monotonic V̇O_2_ from our readings (Olito et al. 2017). All analyses were conducted in R version 3.6.1 (R Development Core Team 2016).

#### Fitness measures: Viability, fertility, and fecundity

We used survival to reproduction (viability), ability to reproduce (fertility), and cumulative reproductive output during the first 20 weeks of the life history (fecundity) as our measures of fitness. Survival and the presence of reproductive structures (ovicells) indicating ability to reproduce were recorded once per week—individuals were considered to be alive if they were still attached to their settlement plate and >10% of the colony contained feeding zooids. The fitness measures of viability and fertility were treated as binary data—individuals that survived to the average reproductive age (viability) and those that reproduced (fertility) were assigned “1,” whereas individuals that died before reproductive age or the onset of reproduction were assigned “0.” Reproductive output (fecundity) was measured as the cumulative number of ovicells throughout the duration of the study, which were counted using a dissecting microscope (×10) once per week, from the onset of reproduction at approximately six weeks post‐outplant. In a previous study of this population, *Bugula* survived up to nine months. Reproductive output during the first four months of the life history reliably predicted lifetime reproductive output (cumulative reproductive output 120 days post‐outplant explained 94% of variance in lifetime reproductive output for this same population; Pettersen et al. 2016). In addition, we measured several life history traits related to fitness over the duration of the study: growth (number of colony bifurcations per week; Keough and Chernoff (1987), longevity (number of days >10% colony remained alive), and age at onset of reproduction (days) up until 140 days post‐outplant.

### ESTIMATES OF SELECTION ON LARVAL MASS AND METABOLIC RATES

We can estimate parameters derived in evolutionary theory to quantify competition‐dependent selection on metabolic rates and provide a relative scope for evolutionary change among environments. First, the opportunity for selection (*I*) describes the amount of variation in relative fitness and determines the maximum potential strength of selection that could occur in a given environment (Schluter 1988). Second, the form of selection acting on any trait, or combination of traits, and whether it changes across environments can be quantified using formal selection analysis (Lande and Arnold 1983). Finally, the intensity of selection (*V)* provides a measure of the overall strength of selection acting on all combinations of traits in each environment irrespective of the particular form (Schluter 1988).

#### Estimating and testing for differences in the opportunity for selection

For each competition environment, we calculated the opportunity for selection (*I*) across environments, $I\;=\sigma_{W}^{2}/{\bar{W}}^{2},$ where $\sigma^{2}$ is variance, and W and$\;\bar{W}\;$describes absolute and mean absolute population fitness, respectively (Crow 1958). We could not assess opportunity for selection in binary (viability and fertility) data and hence only fecundity fitness data were tested. Due to over‐dispersion in our reproductive output count data, we calculated nonparametric bootstrap values using BC*a* intervals within the R package “boot” (Davison and Hinkley 1997; Moorad and Wade 2013; Canty and Ripley 2019).

#### Characterizing selection within and among competition environments

We used a classic multiple regression approach to formally estimate the form of selection on our three traits of interest (larval mass, MR_E_, and MR_L_), for our fitness measures (Lande and Arnold 1983). Using a multiple regression framework allows for standardized and comparable estimates of linear (*β*) and nonlinear (*γ*) selection gradients. To investigate selection further, we split our data into three separate analyses: viability selection (survival to reproduction), fertility selection (ability to reproduce), and fecundity selection (cumulative reproductive output). For viability and fertility selection, individuals that survived/did not survive to reproduce (viability selection), and reproduced/did not reproduce (reproductive selection) were assigned “1” and “0,” respectively, and models were fit using logistic regression in a generalized linear model (Janzen and Stern 1998). Viability and fertility selection coefficients were transformed into linear estimates as per Janzen and Stern (1998). Our reproductive output count data for fecundity selection were over‐dispersed and best fit with generalized linear models using a negative binomial distribution (Dobson et al. 2008). Because only 14 out of 120 individuals in the interspecific competition environment reproduced, we did not have sufficient power to calculate nonlinear coefficients of fecundity selection and hence only linear estimates were calculated in this environment. To prepare data across all competition treatments for selection analysis, we first converted our predictor variables of larval mass, MR_E_, and MR_L_ into units of standard deviation (mean of 0 and standard deviation of 1), and mean‐centered survival and reproductive output by dividing each absolute measure by the mean absolute fitness (Lande and Arnold 1983). Both predictor and response variables were also standardized by “experimental panel,” that is, one of the 10 PVC backing panels on which 36 plates (12 per treatment) were randomly allocated. Although we found no significant interactions between experimental panel and competition environment, or with each of our predictor variables, we wanted to account for spatial variation among panels.

Using a series of nested models, we tested whether there were differences in linear selection, nonlinear selection, or both, between competition environments via a sequential model fitting approach (Draper and John 1988; Chenoweth and Blows 2005). Linear selection on fertility (*χ* ^2^ = 70.064, *df* = 6, *P* < 0.0001) and linear (*χ* ^2^ = 188.504, *df* = 6, *P* < 0.0001) and nonlinear (*χ* ^2^ = 27.820, *df* = 12, *P* < 0.01) fecundity selection (*nocomp* and *intra* comparison only) differed among competition environments. For viability selection, all forms of selection (except for correlational viability selection) showed significant interactions with competition environment (see Results). When selection × competition environment interactions were significant, fitness data were standardized within competition environment, and selection coefficients estimated for each competition treatment separately. Quadratic regression coefficients and their standard errors were doubled before being reported as selection gradients (Stinchcombe et al. 2008).

#### Estimating the intensity of selection

Selection intensity (*V*) is a measure of the overall strength of selection as estimated by the variation in predicted fitness values, and is a function of both selection on, and the distribution of, phenotypes in the population (Schluter 1988). In our study, calculating *V* allows for direct comparison of differences in overall selection on metabolic rate and larval mass between levels of competition, irrespective of what the form of selection is in each competition environment. We calculated the expected fitness (survival to reproduction and reproductive output) for each individual using the full regression model, incorporating linear, quadratic, and correlational regression coefficients within each competition environment for viability and fecundity selection separately (Schluter 1988). *V*_viability_ and *V*_fecundity_ were then calculated as the squared coefficient of variance in the expected fitness values (*V* = CV[expected fitness]^2^). We produced nonparametric bootstrap values for our estimates as described previously (Davison and Hinkley 1997; Moorad and Wade 2013; R Development Core Team 2016).

### COMPETITION‐DEPENDENT COVARIANCE BETWEEN LIFE‐HISTORY AND METABOLIC TRAITS

Metabolic rates are linked with key life history traits that together mediate the pace‐of‐life (Careau et al. 2010; Pettersen et al. 2016; Auer et al. 2018b; Niemelä and Dingemanse 2018; Mathot et al. 2019). To understand how selection on metabolic rates might be mediated through their effects on the pace‐of‐life, we measured three key life history traits in individuals of known metabolic phenotype over 20 weeks post‐outplant.

#### Growth

The relationship among larval mass, MR_E_, and MR_L_ on the growth of colonies (number of bifurcations) was estimated using linear‐mixed effects regressions in a repeated measures framework to determine individual growth rate in the field over time (“lme4” package; Bates et al. 2015). Again, we detected significant three‐way interactions with competition environment (*χ* ^2^ = 15.916, *df* = 18, *P* < 0.001) and hence each competition level was analyzed separately. We used a repeated measures ANCOVA to test for significance of the random effect of experimental panel and its interactions with fixed factors of larval mass, MR_E_, and MR_L_ across the repeated measure of week. We found a significant main effect of experimental panel for *nocomp* and *inter*, which was retained in the final model, but no support for fitting a random‐slopes model (no significant interactions between fixed factors and experimental panel were found).

#### Longevity

Longevity showed an overall bivariate response: while mortality rates were high early in the life history, many individuals survived to the end of the sampling period (140 days). This trend was found in a previous longer term study on the same system (Pettersen et al. 2016). Hence, life span data were fit with a logistic regression: individuals that survived less than or 140 days were assigned “0” and “1,” respectively. The main effects of competition environment, larval mass, MR_E_, and MR_L_ on longevity were tested using a generalized, linear‐mixed effects model (“lme4”; Bates et al. 2015). Because competition environment and its interactions were nonsignificant, we pooled data across all competition environments. All interactions with experimental panel were nonsignificant and were removed from the final model.

#### Age at onset of reproduction

Age at onset of reproduction was also fitted with using logistic regression as per Pettersen et al. (2016). Individuals that developed ovicells <60 days post‐outplant were considered to have an early onset of reproduction and were assigned “1,” whereas individuals noted to develop ovicells after this time (>60) were denoted “0.” Individuals that did not reproduce during the study were excluded from the analysis (see fertility selection described previously). We used generalized, linear‐mixed effects logistic regression as described previously. We found no significant effect of competition environment, or its interactions, thus data were pooled across competition environments. We also found no main effect of experimental panel or any of its interactions, so it was removed from the final model.

## Results

### VARIATION IN REPRODUCTIVE OUTPUT AND THE OPPORTUNITY FOR SELECTION

Competition imposed increasingly negative fitness consequences along a stress gradient, from benign conditions under *nocomp* to highly stressful conditions under *inter*. Average cumulative reproductive output for all individuals (irrespective of phenotype) was highest under no competition, *nocomp* (mean n_ovicells_ ± SE: 361 ± 44); intermediate under intraspecific competition, *intra* (mean n_ovicells_ ± SE: 136 ± 28); and lowest under interspecific competition, *inter* (mean n_ovicells_ ± SE: 26 ± 10). The opportunity for selection (*I*) also increased with competition stress (*I*(*nocomp*) = 0.645, 95% CI, 0.576–0.720; *I*(*intra*) = 0.840, 95% CI, 0.766–0.914; *I*(*inter*) = 0.949, 95% CI, 0.909–0.976) and was 1.5 times smaller in the absence of competition relative to interspecific competition.

### ESTIMATES OF COMPETITION‐DEPENDENT SELECTION GRADIENTS

#### Viability selection

**Figure 1:**
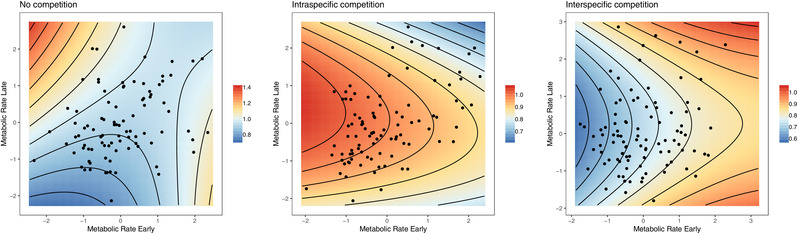
Viability selection surfaces under three competition environments (no competition, intraspecific competition, and interspecific competition) for fitness (survival to reproduction) against metabolic rate early (MR_E_; mJ h^−1^) and metabolic rate late (MR_L_; mJ h^−1^) of *Bugula neritina* settlers. To produce standardized estimates of selection, MR_E_ and MR_L_ were standardized within experimental panel and converted to units of standard deviation (represented by data points) and fitness was mean centered to provide relative fitness. Note that fitness is estimated based on partial regression coefficients for MR_E_ and MR_L_ linear and nonlinear selection gradients.

Our selection analysis revealed significant differences in linear (*χ* ^2^ = 20.575, *df* = 6, *P* = 0.002) and nonlinear (*χ* ^2^ = 44.075, *df* = 12, *P* < 0.0001) viability selection among competition environments. Directional selection was strongest under *nocomp*, with fitness highest for smaller individuals with high MR_L_ (Table 1). Under competition, linear gradients were much weaker and nonsignificant. We did, however, find evidence for significant correlational selection in all competition environments. Across all competition environments, we found negative correlational selection on MR_E_ and MR_L_—individuals with either high MR_E_‐low MR_L_ or vice versa were more likely to survive to reproduce (all competition environments show the same correlational selection coefficients because we found no significant differences in correlational viability selection—see Methods). We also found significant concave selection, but only in the *intra* environment (Fig. 1).

**Table 1:** Viability selection coefficients (± standard error [SE]) for larval mass (μg), metabolic rate early (MR_E_ [mJ/h]), and metabolic rate late (MR_L_ [mJ/h]) with survival to reproduction for *Bugula neritina* colonies across three competition treatments. *β* and *γ* represent linear and nonlinear selection gradients, respectively. Values in bold represent significant results (*P* < 0.05). Shaded boxes show consistent selection gradients among environments

		*γ*		
No competition	*β*	Larval mass	MR_E_	MR_L_
Larval mass	**−0.295 (0.132)**	−0.038 (0.248)	0.036 (0.073)	0.050 (0.073)
MR_E_	0.137 (0.111)		0.152 (0.290)	**−0.080 (0.034)**
MR_L_	**0.247 (0.126)**			0.038 (0.326)

		*γ*		
Intraspecific competition	*β*	Larval mass	MR_E_	MR_L_
Larval mass	−0.113 (0.119)	0.858 (0.606)	0.036 (0.073)	0.050 (0.073)
MR_E_	−0.149 (0.126)		0.024 (0.218)	**−0.080 (0.034)**
MR_L_	−0.012 (0.125)			**−0.288 (0.160)**

		*γ*		
Interspecific competition	*β*	Larval mass	MR_E_	MR_L_
Larval mass	0.054 (0.091)	0.198 (0.174)	0.036 (0.073)	0.050 (0.073)
MR_E_	0.102 (0.092)		−0.004 (0.176)	**−0.080 (0.034)**
MR_L_	0.013 (0.081)			0.132 (0.136)

#### Fertility selection

Competition affected the probability of reproducing over the first 20 weeks of the life history—individuals under *nocomp* and *intra* were more likely to reproduce than individuals under *inter* (*χ* ^2^ = 72.389, *df* = 2, *P* < 0.0001). We found significant differences in linear (*χ* ^2^ = 70.064, *df* = 8, *P* < 0.0001) but not nonlinear (*χ* ^2^ = 12.415, *df* = 12, *P* = 0.413) fertility selection across competition environments. Under *nocomp*, linear fertility selection tended to favor individuals with low metabolic rates (although not significantly), whereas under *intra* and *inter*, selection favored higher MR_E_ and MR_L_, respectively (*β_MRE_* ± SE = 0.144 ± 0.062; *β_MRL_* ± SE = 0.120 ± 0.046). Across *nocomp* and *intra*, we found evidence for negative quadratic selection on larval mass only (*γ_Mass,Mass_* ± SE = 0.212 ± 0.058). All linear and nonlinear fertility selection coefficients are provided in Table S1.

#### Fecundity selection

**Figure 2:**
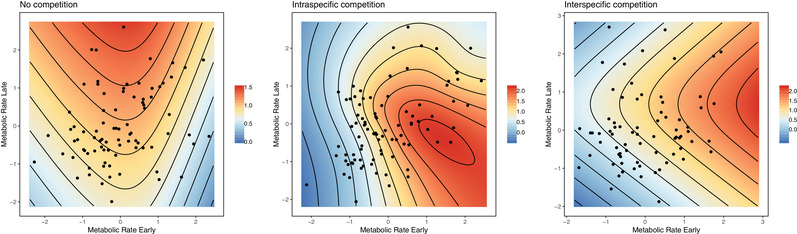
Fecundity selection surfaces under three competition environments (no competition, intraspecific competition, and interspecific competition) for fitness (total cumulative reproductive output over first 20 weeks post‐outplant) against metabolic rate early (MR_E_; mJ h^−1^) and metabolic rate late (MR_L_; mJ h^−1^) of *Bugula neritina* settlers. To produce standardized estimates of selection, MR_E_ and MR_L_ were standardized within experimental panel and converted to units of standard deviation (represented by data points) and fitness was mean centered to provide relative fitness. Note that fitness is estimated based on partial regression coefficients for MR_E_ and MR_L_ linear and nonlinear selection gradients. Due to insufficient fecundity data, interspecific competition selection surface was fit using linear selection coefficients only.

We found both linear (all competition environments; *χ* ^2^ = 188.504, *df* = 6, *P <* 0.0001) and nonlinear (*nocomp* and *intra* only; *χ* ^2^ = 27.820, *df* = 12, *P* < 0.01) fecundity selection varied with competition environment. Under *nocomp*, intermediate MR_E_ showed highest reproductive output (Table 2). Under *intra*, we found evidence for directional selection for high MR_E_, and negative quadratic selection on larval mass, MR_E_ and MR_L_, where individuals with high MR_E_ and intermediate MR_L_ were favored. We found negative correlational selection under *intra*—individuals with either high MR_E_/low MR_L_ or vice versa showed highest fecundity. Due to directional selection for high metabolic phenotypes under *inter*, reproductive output was greatest for individuals with both high MR_E_ and MR_L_ and lowest for individuals with low metabolic rates (Fig. 2).

**Table 2:** Fecundity selection coefficients (± standard error [SE]) for larval mass (μg), metabolic rate early (MR_E_ [mJ/h]), and metabolic rate late (MR_L_ [mJ/h]) with reproductive output for *Bugula neritina* colonies across three competition treatments. *β* and *γ* represent linear and nonlinear selection gradients, respectively. Values in bold represent significant results (*P* < 0.05)

		*γ*		
No competition	*β*	Larval mass	MR_E_	MR_L_
Larval mass	−0.153 (0.198)	−0.170 (0.340)	−0.213 (0.207)	−0.170 (0.214)
MR_E_	−0.001 (0.164)		**−0.404 (0.236)**	0.098 (0.172)
MR_L_	0.187 (0.156)			−0.182 (0.254)

		*γ*		
Intraspecific competition	*β*	Larval mass	MR_E_	MR_L_
Larval mass	−0.072 (0.223)	**−0.376 (0.272)**	−0.088 (0.235)	0.058 (0.293)
MR_E_	**0.450 (0.226)**		**−0.712 (0.352)**	**−0.521 (0.217)**
MR_L_	−0.017 (0.225)			**−0.510 (0.312)**

		*γ*		
Interspecific competition	*β*	Larval mass	MR_E_	MR_L_
Larval mass	**−0.566 (0.349)**			
MR_E_	**0.563 (0.349)**			
MR_L_	**0.209 (0.315)**			

*Note*. Fecundity data were too sparse to estimate *γ* for interspecific competition.

### ESTIMATING THE INTENSITY OF SELECTION

The intensity of viability selection (*V*_viability_) was significantly higher in the absence of competition—*V*_viability_ was over eight orders of magnitude higher under *nocomp* (*V*_viability_ = 0.261; CI, 0.125–0.360) than under either *intra* (*V*_viability_ = 0.037; CI, 0.017–0.090) and *inter* (*V*_viability_ = 0.031; CI, 0.012–0.078). For individuals that survived to reproductive age, we did not detect any significant differences in the intensity of either fertility selection (*V*_fertility_) or intensity of fecundity selection (*V*_fecundity_) across competition environments.

### COMPETITION‐DEPENDENT COVARIANCE AMONG LARVAL MASS, METABOLIC RATES, AND LIFE‐HISTORY TRAITS

#### Growth

**Figure 3:**
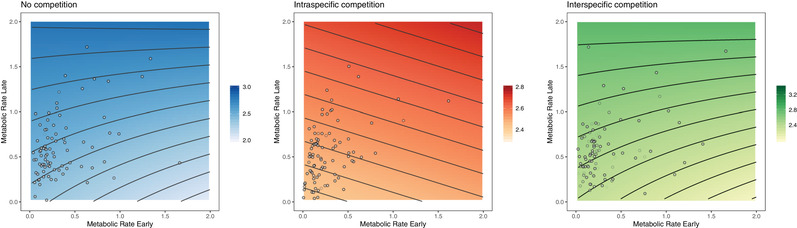
Linear mixed effects models for predicted growth rate (number of bifurcations over the first 20 weeks post‐output) plotted against metabolic rate early (MR_E_; mJ h^−1^) and metabolic rate late (MR_L_; mJ h^−1^) in *Bugula neritina* settlers across three competition environments (blue, no competition; red, intraspecific competition; green, interspecific competition). For illustrative purposes, “week” has been held constant at 5 weeks post‐outplant and MR_E_ and MR_L_ are standardized by “experimental panel” as shown by data points (both terms were included in the final mixed effects model; Table S2).

MR_E_ and MR_L_ had significant, but context‐dependent effects on growth. Colonies consistently increased in size throughout the first 20 weeks post‐outplant, but growth was highest under *nocomp* and lowest under *inter*. Overall, higher metabolic rates were associated with faster growth in both competition environments—and this was most evident under *inter* (Fig. 3). Interestingly in *nocomp*, growth was slowest for individuals with both high MR_E_ and low MR_L_, despite a strong positive interaction between the two metabolic rates (Table S2; Fig. 3).

#### Longevity

**Figure 4:**
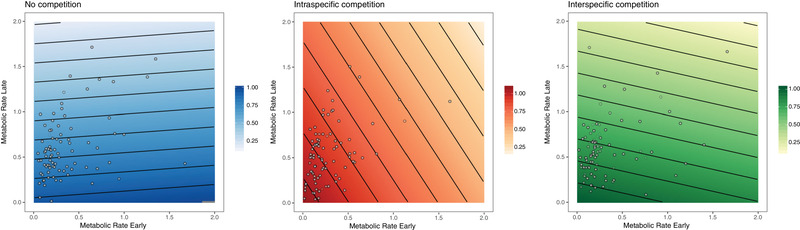
Logistic regression models for predicted longevity (probably of surviving >140 days) plotted against metabolic rate early (MR_E_; mJ h^−1^) and metabolic rate late (MR_L_; mJ h^−1^) in *Bugula neritina* settlers across three competition environments (blue, no competition; red, intraspecific competition; green, interspecific competition). Data points represent raw MR_E_ and MR_L_ data.

We found significant main effects of competition environment, MR_E_, and MR_L_ on longevity—overall, colonies under *inter* were shorter lived than those in the *intra* or *nocomp* environments (mean ± SE; *nocomp*: 132 ± 2.89 days, *intra*: 129 ± 3.11 days, *inter*: 101 ± 4.69 days). Across all competition environments, however, individuals with lower MR_E_ and lower MR_L_ lived longer (Fig. 4). This relationship was consistent among experimental panels and no significant interactions among fixed or random effects were found (Table S3).

#### Age at onset of reproduction

The onset of reproduction occurred between 28 and 126 days after being deployed into the field (mean ± SE = 65.29 ± 1.73 days). Individuals with higher MR_L_ began reproducing sooner—we found a significant positive relationship between MR_L_ and the probability of early onset of reproduction, and this was consistent across all competition environments (Table S4).

## Discussion

Competition changed the strength and form of selection on metabolic rates in the field. Survival, fertility, and fecundity were lowest under interspecific competition and highest in the absence of competition. Fertility and fecundity (but not viability) selection on metabolic rates changed along a stress gradient—when competition was absent, weak quadratic selection favored intermediate phenotypes, whereas strong directional and quadratic selection favored higher metabolic rates under competition. Previous studies have shown that context‐ and resource‐dependent selections on metabolic rates vary across space and time (Boratyński and Koteja 2010; Zub et al. 2014; Auer et al. 2018a). The natural environment for *Bugula neritina* is a mosaic of competitor‐free, intra‐ and interspecific competition, with individuals from the same brood potentially experiencing very different environments (Chang and Marshall 2016). Our competition treatments reflect the scale of this variability—individual settlers were separated by only a few centimeters, yet experienced distinct selection regimes. We find evidence that metabolism mediates a trade‐off between competition and colonization via the pace‐of‐life—high metabolism individuals withstood competition, but low metabolism individuals are likely to live longer in newly colonized, competitor‐free environments. Although selection on metabolic rates was strong, its context‐dependent nature will likely hamper its capacity to purge variance in metabolism.

Competition tended to favor higher metabolic rates, perhaps because they also covaried with faster life histories. Under interspecific competition, individuals with higher metabolic rates were more likely to survive, more likely to reproduce, and were more fecund upon the onset of reproduction. Under interspecific competition, higher metabolism covaried with a faster pace‐of‐life (i.e., faster growth, shorter life span (MR_E_ and MR_L_), and earlier onset of reproduction (MR_L_)). Higher metabolic rates are often associated with a faster pace‐of‐life in stressful environments. For example, individuals with higher metabolic rates grow faster and display more aggression—exerting dominance to secure access to food, mates, and territory (Reid et al. 2011; Le Galliard et al. 2013; Auer et al. 2018b). In *Bugula*, higher metabolic rates may increase feeding rates and energy acquisition, allowing individuals to emerge from the canopy of other competing individuals or species sooner, to reach resources such as food and oxygen. Yet, higher metabolic rates also come with the cost of a shorter life span, as shown in our study and in others (Bochdansky et al. 2005). A negative covariance between lifespan and metabolic rate could be a result of increased oxidative stress associated with higher metabolism (Dowling and Simmons 2009), yet there is also evidence to the contrary (Salin et al. 2015); hence, any causal explanation is likely to be both complex as well as system and context dependent. Given the intensity of selection under competition, where life expectancies are lower, the selective advantage of faster growth rates and earlier reproduction is likely to compensate for the reduced longevity associated with higher metabolic rates.

Given that we observe strong directional selection for higher metabolic rates in most environments, why are lower metabolic rates not purged from the population? We find cryptic benefits of low metabolic phenotypes (particularly for MR_E_) in the absence of competition—for example, low‐metabolism individuals had a higher probability of living for longer than high‐metabolism individuals. Because we ended our experiments before low‐metabolism individuals in competition‐free environments had perished, we underestimated their fitness—these individuals would have continued to reproduce long after high‐metabolism individuals had died. As such, low‐metabolism individuals likely have a fitness advantage over high‐metabolism individuals when competition is absent. Metabolism therefore appears to mediate a competition‐colonization trade‐off via pace‐of‐life effects in our system. High‐metabolism individuals grow more and reproduce sooner before dying earlier—a phenotype that confers higher fitness when competition is intense. Meanwhile, low‐metabolism individuals grow slowly, but live longer, suffering reduced fitness when competition is strong, but gaining higher fitness when they colonize competition‐free environments. Such trade‐offs are known to maintain variation (Kisdi and Geritz 2003). Importantly, this is not the only mechanism by which variation in metabolic rate will be maintained—we also found ubiquitous negative correlational selection on different metabolic rates, particularly in high‐competition environments. Negative correlational selection will act to increase negative covariance between metabolic traits, hampering the capacity of strong positive directional selection to increase trait values of both simultaneously.

In our system, competition‐free habitat is rare and ephemeral in nature—hence, competitive environments should impose strong selection on metabolic rates. However, fitness payoffs for individuals with lower metabolic rates colonizing rare, competition‐free environments are considerable (they have much higher fecundities). Although rare, massive reproductive payoffs in competition‐free habitats might therefore be sufficient to maintain low metabolic rate phenotypes, particularly because selection was most intense in competition‐free environments. Thus, selection may be unable to purge low metabolic rates if these individuals are occasionally able to invade free space (Courbaud et al. 2012). Ultimately, countervailing selection pressures acting on survival and reproduction, and the considerable fitness benefits of lower metabolic rates under competition‐free environments, however rare, may contribute to maintaining variance in metabolic rates (Wadgymar et al. 2017).

We found negative correlational viability selection opposed a positive, albeit weak, phenotypic correlation between MR_E_ and MR_L_. If phenotypic correlations are representative of underlying positive genetic correlations between MR_E_ and MR_L_, then genetic constraints may limit the efficacy of this negative correlational selection (Blows 2007; Pettersen et al. 2016). Our findings highlight the importance of measuring multiple metabolic rates—estimates of selection on either MR_E_ or MR_L_ in isolation fail to account for any underlying covariance between correlated characters that may override the effects of univariate selection (Hansen et al. 2019). We show that metabolic rate is not a single trait, but varies across ontogeny and importantly, selection “perceives” and distinguishes between these traits. Thus, measures of multivariate selection are necessary to reveal the full picture of selection acting on metabolic rates.

How does competition alter the process and outcome of selection? Competition decreased mean individual fitness, yet increased variation in fecundity. Competition also reduced total viability and fecundity selection intensity. Although competition environments offered greater potential for selection, overall selection on the suite of traits measured (larval mass, MR_E_, and MR_L_), and hence variation in predicted fitness, was reduced under competition. Accordingly, although the opportunity for selection increased, the intensity of selection decreased under competition. Others have argued that higher stress does not always translate into increased strength of selection (Agrawal and Whitlock 2010); our results support this sentiment.

Competition dramatically altered selection on metabolic phenotypes. Many factors act to hamper the purging of any one metabolic phenotype in our system. Because metabolism is linked with the pace‐of‐life, it mediates a competition‐colonization trade‐off—this trade‐off in turn generates extremely variable selection within the population. Meanwhile, even within a single environment, ubiquitous negative correlational selection hampers the capacity of strong directional selection to increase trait values. In light of these findings, intrapopulation variation in metabolic rate, rather than representing a challenge to theory, seems almost inevitable.

## AUTHOR CONTRIBUTIONS

DJM and CRW conceived of the study. All authors developed the study design. AKP collected the data. AKP, DJM, and MDH analyzed the data. AKP wrote the first draft of the manuscript. All authors contributed substantially to revisions.

## DATA ARCHIVING

Upon acceptance of the manuscript, all data and code supporting the results will be archived in a public depository (https://doi.org/10.5061/dryad.ksn02v71d).

Associate Editor: R. Snook

## Supporting information


**Table S1**. Fertility selection coefficients (± standard error; SE) for Larval mass (μg), Metabolic rate early (MRE; mJh^‐1^), and Metabolic rate late (MRL; mJ h^‐1^) with ability to reproduce for *Bugula neritina* colonies across three competition treatments.
