# Supplementary material for: Metabolic rate, context‐dependent selection, and the competition‐colonization trade‐off
Source: Evol Lett. 2020 Jun 12;4(4):333–44. doi: 10.1002/evl3.174 (PMC7403701; doi:10.1002/evl3.174)
Supplement: Supplementary file 1 — Table S1. Fertility selection coefficients (± standard error; SE) for Larval mass (μg), Metabolic rate early (MRE; mJh‐1), and Metabolic rate late (MRL; mJ h‐1) with ability to reproduce for Bugula neritina colonies across three competition treatments. [file EVL3-4-333-s001.docx]

**Supporting information**

Title: Metabolic rate, context-dependent selection, and the competition-colonisation trade-off

Short running title: Competition and selection on metabolic rates

Type of article: Letter

Author affiliation: Amanda K. Pettersen^1,2*^, Matthew D. Hall^1^, Craig R. White^1^, Dustin J. Marshall^1^

^1^ School of Biological Sciences/Centre for Geometric Biology, Monash University, Melbourne, Australia

^2^ Department of Biology, Lund University, Lund, Sweden

*Corresponding author: Department of Biology, Lund University, Sölvegatan 37, 22362, Lund, Sweden. Phone: +46 72-190 55 48. Email: amanda.pettersen@biol.lu.se

Keywords: intra-specific competition, inter-specific competition, metabolism, fitness, pace-of-life, viability, fertility, fecundity, growth, longevity, reproduction, larval size

**Tables**

Table S1. Fertility selection coefficients (± standard error; SE) for Larval mass (μg), Metabolic rate early (MRE; mJh^-1^), and Metabolic rate late (MRL; mJ h^-1^) with ability to reproduce for *Bugula neritina* colonies across three competition treatments. (*β* and *γ* represent linear and nonlinear selection gradients, respectively. Values in bold represent significant results (*p* < 0.05). Shaded boxes show consistent selection gradients among environments.

| No competition | *β* | *γ* | | |
| --- | --- | --- | --- | --- |
|  |  | Larval mass | MR_E_ | MR_L_ |
| Larval mass | 0.027 (0.166) | **0.212 (0.058)** | -0.103 (0.074) | -0.062 (0.084) |
| MR_E_ | -0.026 (0.134) |  | -0.178 (0.062) | 0.099 (0.071) |
| MR_L_ | -0.010 (0.130) |  |  | -0.090 (0.059) |
| Intraspecific competition | *β* | *γ* | | |
|  |  | Larval mass | MR_E_ | MR_L_ |
| Larval mass | -0.042 (0.116) | **0.212 (0.058)** | -0.103 (0.074) | -0.062 (0.084) |
| MR_E_ | **0.144 (0.062)** |  | -0.178 (0.062) | 0.099 (0.071) |
| MR_L_ | -0.051 (0.116) |  |  | -0.090 (0.059) |
| Interspecific competition | *β* | *γ* | | |
|  |  | Larval mass | MR_E_ | MR_L_ |
| Larval mass | -0.062 (0.149) | **0.212 (0.058)** | -0.103 (0.074) | -0.062 (0.084) |
| MR_E_ | 0.066 (0.140) |  | -0.178 (0.062) | 0.099 (0.071) |
| MR_L_ | **0.120 (0.046)** |  |  | -0.090 (0.059) |

Table S2. Linear mixed effects repeated measures regression for growth rate (change in number of bifurcations across weeks) post-outplant in *Bugula neritina*. Results from log-likelihood tests, values in bold represent significant results (*p-value* <0.05).

| Environment | No competition | | | | Intraspecific competition | | | | Interspecific competition | | | |
| --- | --- | --- | --- | --- | --- | --- | --- | --- | --- | --- | --- | --- |
|  | χ^2^ | df | *p-value* | Estimate (±SE) | χ^2^ | df | *p-value* | Estimate (±SE) | χ^2^ | df | *p-value* | Estimate (±SE) |
| *Fixed effects* |  |  |  |  |  |  |  |  |  |  |  |  |
| Time (week) | 611.231 | 1 | **<0.001** | 0.348  (±0.007) | 492.535 | 1 | **<0.001** | 0.040  (±0.075) | 222.368 | 1 | **<0.001** | 0.189  (±0.008) |
| Larval mass | 2.496 | 1 | 0.114 |  | 4.037 | 1 | **0.045** | 0.213  (±0.080) | 2.384 | 1 | 0.123 |  |
| MR_E_ | 232.234 | 1 | **<0.001** | -0.508  (±0.331) | 187.154 | 1 | **<0.001** | 1.281  (±1.347) | 131.599 | 1 | **<0.001** | -0.764  (±0.284) |
| MR_L_ | 226.849 | 1 | **<0.001** | 0.330  (±0.263) | 184.671 | 1 | **<0.001** | 3.579  (±1.499) | 208.244 | 1 | **<0.001** | -0.248  (±0.190) |
| Time x Larval mass | 1.060 | 1 | 0.303 |  | 3.484 | 1 | 0.062 |  | 3.000 | 1 | 0.083 |  |
| Time x MR_E_ | 0.921 | 1 | 0.337 |  | 4.806 | 1 | 0.028 |  | 0.981 | 1 | 0.322 |  |
| Time x MR_L_ | 1.135 | 1 | 0.287 |  | 0.605 | 1 | 0.437 |  | 1.275 | 1 | 0.259 |  |
| Larval mass x MR_E_ | 0.854 | 1 | 0.355 |  | 8.740 | 1 | **0.003** | -0.041  (±0.127) | 0.004 | 1 | 0.950 |  |
| Larval mass x MR_L_ | 1.106 | 1 | 0.293 |  | 21.056 | 1 | **<0.001** | -0.360  (±0.134) | 2.972 | 1 | 0.085 |  |
| MR_E_ x MR_L_ | 5.997 | 1 | **0.014** | 0.580  (±0.386) | 0.170 | 1 | 0.680 |  | 3.918 | 1 | **0.048** | 0.973  (±0.296) |
| Time x Larval mass x MR_E_ | 1.597 | 1 | 0.206 |  | 0.241 | 1 | 0.623 |  | 0.077 | 1 | 0.781 |  |
| Time x Larval mass x MR_L_ | 0.622 | 1 | 0.430 |  | 4.506 | 1 | **0.034** | -0.021  (±0.011) | 0.500 | 1 | 0.480 |  |
| Time x MR_E_ x MR_L_ | 0.028 | 1 | 0.867 |  | 0 | 1 | 1 |  | 0.991 | 1 | 0.609 |  |
| Larval mass x MR_E_ x MR_L_ | 0.028 | 1 | 0.867 |  | 0 | 1 | 1 |  | 0.991 | 1 | 0.319 |  |
| Time x Larval mass x MR_E_ x MR_L_ | 3.549 | 1 | 0.060 |  | 0.239 | 1 | 0.625 |  | 3.428 | 1 | 0.064 |  |
| *Random effect* |  |  |  |  |  |  |  |  |  |  |  |  |
| Experimental panel | 14.833 | 1 | **<0.001** |  | 25.861 | 1 | **<0.001** |  | 3.315 | 1 | 0.069 |  |

Table S3. Logistic generalised linear mixed effects regression for longevity (individuals that survived less than or more than 140 days were assigned “0” and “1”, respectively) in *Bugula neritina*. Results from log-likelihood tests, values in bold represent significant results (*p-value* <0.05).

|  | χ^2^ | df | *p-value* | Estimate |
| --- | --- | --- | --- | --- |
| *Fixed effects* |  |  |  |  |
| Environment | 22.109 | 2 | **<0.001** |  |
| Larval mass | 1.664 | 1 | 0.197 |  |
| MR_E_ | 12.659 | 1 | **<0.001** | -0.785 |
| MR_L_ | 12.797 | 1 | **<0.001** | -1.625 |
| Environment x Larval mass | 0.845 | 2 | 0.655 |  |
| Environment x MR_E_ | 4.157 | 2 | 0.125 |  |
| Environment x MR_L_ | 1.047 | 2 | 0.592 |  |
| Larval mass x MR_E_ | 0.173 | 1 | 0.678 |  |
| Larval mass x MR_L_ | 0.121 | 1 | 0.290 |  |
| MR_E_ x MR_L_ | 0.343 | 1 | 0.558 |  |
| Environment x Larval mass x MR_E_ | 3.049 | 2 | 0.217 |  |
| Environment x Larval mass x MR_L_ | 2.240 | 2 | 0.326 |  |
| Environment x MR_E_ x MR_L_ | 0.794 | 2 | 0.851 |  |
| Larval mass x MR_E_ x MR_L_ | 0.784 | 1 | 0.699 |  |
| Environment x Larval mass x MR_E_ x MRE_L_ | 1.026 | 2 | 0.599 |  |
| *Random effect* |  |  |  |  |
| Experimental panel | 0.3673 | 1 | 0.544 |  |

Table S4. Logistic generalised linear mixed effects regression for age at onset of reproduction (number of days post-outplant when ovicells developed) in *B. neritina*. Individuals were assigned either “1” for early (<60 days) or “0” for late (>60 days) onset of reproduction. Results from log-likelihood tests, values in bold represent significant results (*p-value* <0.05).

|  | χ^2^ | df | *p-value* | Estimate |
| --- | --- | --- | --- | --- |
| *Fixed effects* |  |  |  |  |
| Environment | 0.294 | 2 | 0.863 |  |
| Larval mass | 0.914 | 1 | 0.339 |  |
| MR_E_ | 0.914 | 1 | 0.339 |  |
| MR_L_ | 11.321 | 1 | **<0.001** | 0.475 |
| Environment x Larval mass | 1.177 | 2 | 0.974 |  |
| Environment x MR_E_ | 1.611 | 2 | 0.498 |  |
| Environment x MR_L_ | 2.389 | 2 | 0.296 |  |
| Larval mass x MR_E_ | 0.427 | 2 | 0.261 |  |
| Larval mass x MR_L_ | 0.728 | 1 | 0.312 |  |
| MR_E_ x MR_L_ | 0.655 | 1 | 0.309 |  |
| Environment x Larval mass x MR_E_ | 3.407 | 2 | 0.217 |  |
| Environment x Larval mass x MR_L_ | 0.871 | 2 | 0.647 |  |
| Environment x MR_E_ x MR_L_ | 2.157 | 2 | 0.540 |  |
| Larval mass x MR_E_ x MR_L_ | 0.031 | 1 | 0.861 |  |
| Environment x Larval mass x MR_E_ x MRE_L_ | 2.195 | 2 | 0.334 |  |
| *Random effects* |  |  |  |  |
| Experimental panel | 0.006 | 1 | 0.939 |  |


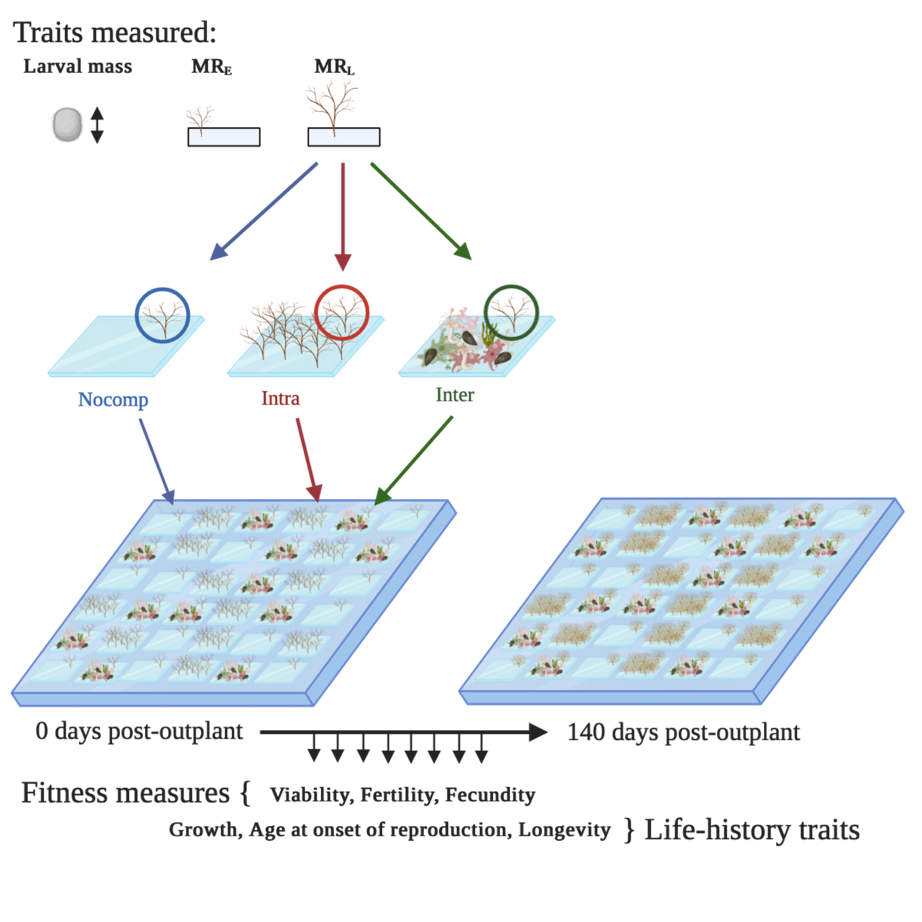


Figure S1. Schematic showing experimental setup. Larval mass, and two metabolic rates: metabolic rate early (2 hours post-settlement; MR_E_) and metabolic rate late (24 hours post-settlement; MR_L_) were measured for 360 individual *Bugula neritina*. Each focal individual (circled) was then was glued onto a labelled PVC plate (55 x 55 x 3mm) and assigned to one of three competition treatments: a no competition environment (“Nocomp”), an intraspecific environment (“Intra”): a single focal individual settler was glued onto a plate among eight individual conspecific settlers of the same age, or an interspecific environment (“Inter”): a single focal individual settler was glued into a pre-established, subtidal community. 36 plates, comprising of a combination of the three competition treatments were then attached to one of 10 backing panels suspended in the subtidal at approximately 1.5m depth. Individuals were monitored for fitness measures of viability (survival), fertility (onset of reproduction) and fecundity (number of ovicells), as well we life-history traits (growth, age at onset of reproduction, longevity) approximately once per week until 140 days post-outplant.
